# Changes in the frequencies of *Plasmodium falciparum dhps* and *dhfr* drug-resistant mutations in children from Western Kenya from 2005 to 2018: the rise of *Pfdhps* S436H

**DOI:** 10.1186/s12936-020-03454-8

**Published:** 2020-10-22

**Authors:** M. Andreína Pacheco, Kristan A. Schneider, Qiuying Cheng, Elly O. Munde, Caroline Ndege, Clinton Onyango, Evans Raballah, Samuel B. Anyona, Collins Ouma, Douglas J. Perkins, Ananias A. Escalante

**Affiliations:** 1 Biology Department/Institute of Genomics and Evolutionary Medicine (iGEM), Temple University, Philadelphia, PA USA; 2 Department of Applied Computer and Biosciences, University of Applied Sciences Mittweida, Technikumplatz, Mittweida, Germany; 3 Center for Global Health, University of New Mexico Health Sciences Center, Albuquerque, NM USA; 4 University of New Mexico-Kenya Global Health Programs, Kisumu, Siaya Kenya; 5 Department of Clinical Medicine, School of Health Sciences, Kirinyaga University, Kerugoya, Kenya; 6 Department of Biomedical Sciences and Technology, Maseno University, Maseno, Kenya; 7 Department of Medical Laboratory Sciences, School of Public Health, Biomedical Sciences and Technology, Masinde Muliro University of Science and Technology, Kakamega, Kenya; 8 Department of Medical Biochemistry, School of Medicine, Maseno University, Maseno, Kenya

**Keywords:** Drug resistance genes, *Dhfr*, *Dhps*, *k13* gene, SP resistance, *Plasmodium falciparum*

## Abstract

**Background:**

Sulfadoxine-pyrimethamine (SP) is the only anti-malarial drug formulation approved for intermittent preventive treatment in pregnancy (IPTp). However, mutations in the *Plasmodium falciparum dhfr* (*Pfdhfr*) and *dhps* (*Pfdhps*) genes confer resistance to pyrimethamine and sulfadoxine, respectively. Here, the frequencies of SP resistance-associated mutations from 2005 to 2018 were compared in samples from Kenyan children with malaria residing in a holoendemic transmission region.

**Methods:**

Partial sequences of the *Pfdhfr* and *Pfdhps* genes were amplified and sequenced from samples collected in 2005 (n = 81), 2010 (n = 95), 2017 (n = 43), and 2018 (n = 55). The frequency of known mutations conferring resistance to pyrimethamine and sulfadoxine were estimated and compared. Since artemisinin-based combination therapy (ACT) is the current first-line treatment for malaria, the presence of mutations in the propeller domain of *P. falciparum kelch13* gene (*Pfk13*) linked to ACT-delayed parasite clearance was studied in the 2017/18 samples.

**Results:**

Among other changes, the point mutation of *Pfdhps* S436**H** increased in frequency from undetectable in 2005 to 28% in 2017/18. Triple *Pfdhfr* mutant allele (C**IRN**I) increased in frequency from 84% in 2005 to 95% in 2017/18, while the frequency of *Pfdhfr* double mutant alleles declined (allele C**I**C**N**I from 29% in 2005 to 6% in 2017/18, and CN**RN**I from 9% in 2005 to undetectable in 2010 and 2017/18). Thus, a multilocus *Pfdhfr*/*Pfdhps* genotype with six mutations (**HGE**AA/C**IRN**I), including *Pfdhps* S436**H**, increased in frequency from 2010 to 2017/18. Although none of the mutations associated with ACT-delayed parasite clearance was observed, the *Pfk13* mutation A578S, the most widespread *Pfk13* SNP found in Africa, was detected in low frequency (2.04%).

**Conclusions:**

There were changes in SP resistance mutant allele frequencies, including an increase in the *Pfdhps* S436**H**. Although these patterns seem consistent with directional selection due to drug pressure, there is a lack of information to determine the actual cause of such changes. These results suggest incorporating molecular surveillance of *Pfdhfr*/*Pfdhps* mutations in the context of SP efficacy studies for intermittent preventive treatment in pregnancy (IPTp).

## Background

Despite a worldwide decline, malaria remains a significant and resilient global health problem. Approximately 228 million cases and 405,000 associated deaths were reported globally in 2018; of those, more than 90% of the malaria morbidity and mortality occurred in Africa [1]. *Plasmodium falciparum* is the most prevalent malaria parasite in the African continent, accounting for 99.7% of the estimated cases in sub-Saharan Africa. Pregnant women and children under 5 years of age are the most vulnerable groups and account for 67% of all malaria deaths worldwide. The interventions available to mitigate the adverse effects of malaria during pregnancy include intermittent preventive treatment in pregnancy (IPTp), insecticide-treated bed-nets (ITNs), and case management [2, 3]. Currently, sulfadoxine-pyrimethamine (SP) is the only anti-malarial drug formulation approved for use in IPTp [3]. The SP drug inhibits the enzymes dihydrofolate reductase (DHFR) and dihydropteroate synthase (DHPS). These enzymes are involved in the folate pathway of nucleic acid synthesis [4, 5]. However, mutations in the parasite genes *dhfr* (*Pfdhfr*) and *dhps* (*Pfdhps*) confer different degrees of resistance to pyrimethamine and sulfadoxine, respectively [4–8]. Specifically, there are four-point mutations in *Pfdhfr* (N51I, C59R, S108N, and I164L) and five in *Pfdhps* (S436A/F, A437G, K540E, A581G, and A613S/T) [6, 7, 9–13].

Due to the increasing SP resistance and pervasive chloroquine resistance, the World Health Organization (WHO) recommended artemisinin-based combination therapy (ACT) as first-line treatment for uncomplicated malaria in most endemic countries [3, 14]. However, ACT is still not approved for the prevention of malaria in pregnant women due to the absence of adequate safety data [3, 15]. Thus, SP remains the only drug used for IPTp and is being considered for intermittent preventive treatment in infants (IPTi) [15–17].

Several studies in Kenya have shown an association between the *Pfdhfr* triple mutant (N51I, C59R, S108N) combined with the *Pfdhps* double mutant (A437G, K540E), and resistance to SP in vivo [13, 18]. Even after SP was no longer the first-line drug in Kenya as of 2004, the *Pfdhfr/Pfdhps* quintuple mutant genotype (N51I, C59R, S108N/A437G, K540E) continued to be prevalent [13]. Given that SP remains in use for IPTp, is considered as a possible ACT partner drug, and is a candidate for IPTi, the frequencies of SP resistance-associated mutations were investigated in samples collected from pediatric malaria patients in Siaya (Western Kenya) during three periods: 2005, 2010, and 2017/18. Among other well-known mutations associated with SP resistance, the change in frequency of a novel mutation identified in *Pfdhps* (S436**H**) [19] was also estimated. In addition, to obtain a more comprehensive picture of the mutations related with anti-malarial drug resistance, the presence of mutations linked to the delayed parasite clearance phenotype against artemisinin-based combinations were assessed by studying the polymorphism in the propeller domain of the *P. falciparum kelch13* gene (*Pfk13*) in a group of samples collected in 2017/18 [1, 20].

## Methods

### Study sites, sample collection, and DNA isolation

Samples were initially collected as part of an immunoepidemiologic study approved by the Ethics Committee of the Kenya Medical Research Institute, the University of New Mexico Institutional Review Board, the Los Alamos National Laboratory (LANL) Institutional Review Board, and the Maseno University Ethics Review Committee. The study was conducted at Siaya County Referral Hospital (SCRH), a holoendemic *P. falciparum* transmission region in Western Kenya. Details of the study design and study area have been previously published [21]. Individuals inhabiting the study area are predominantly from the Luo ethnic group (> 96%). Children (primarily aged < 12 months), who presented at the paediatric ward for their first 'hospital contact' (for any reason), were identified and screened for malaria parasites. Children were enrolled in the cohort studies unless they met any of the following exclusion criteria: positive blood smears with non-*P. falciparum* species, previous hospitalization (for any reason), documented or reported use of anti-malarial therapy 2 weeks prior to enrollment, and/or cerebral malaria diagnosis (though rare in this study area). Informed written consent was obtained from the parents/legal guardians of all participating children. All children were treated with standard anti-malarials approved at the time following the local guidelines (Coartem™: artemether and lumefantrine). About 2 mL of venous blood was obtained from each study participant at enrollment or visit and used for genotyping analysis.

To explore changes in mutations linked to drug resistance, *P. falciparum* genomic DNA was extracted from 200 µl of 81 blood samples collected in 2005 and 95 blood samples collected in 2010 using QIAamp DNA Micro Kit (Qiagen, GmbH, Hilden, Germany). For samples collected in 2017/18, genomic DNA was extracted using Direct-zol DNA/RNA miniprep kit (Zymo Research, Tustin, CA, USA) from 200 µL aliquots of each of the 98 blood samples that were mixed with Tri reagent (Thermo Fisher Scientific, Waltham, MA, USA).

### Genotyping analysis of *P. falciparum* drug resistance genes and *Pfk13* gene

Drug resistance genes and *kelch13* gene (*Pfk13*) of *P. falciparum* were amplified by polymerase chain reaction (PCR). DNA samples were genotyped for mutations at: (1) *P. falciparum* hydroxymethyldihydropterin pyrophosphokinase-dihydropteroate synthase gene (*Pfdhps*) codons 436, 437, 540, 581, and 613; (2) *P. falciparum* dihydrofolate reductase-thymidylate synthase gene (*Pfdhfr-ts*) codons 51, 59, 108, and 164; and (3) the propeller domain coding region (720 bp) of *Pfk13* (with an open reading frame of 2,181 bp in length). *Pfk13* mutation analysis was only performed for samples collected in the period of 2017/18. A fragment of 750 out of 2418 bp for *Pfdhps* and a fragment of 1688 out of 1827 bp for *Pfdhfr* were amplified. Sequences of PCR primers used in this study were: (1) for *Pfdhps*, 5′-GAT ATA TGT ATT AAA AGA TAG AAT TTC-3′ (forward) and 5′-CTT GTC TTT CCT CAT GTA ATT C-3′ (reverse); (2) for *Pfdhfr*, 5′-GCM ATA TGT GCA TGT TGT AAR G-3′ (forward) and 5′-GCC ATA TCC ATT KAA ATT TTW TC-3′ (reverse); and (3) for *Pfk13*, 5′-GAT AAA CAA GGA AGA ATA TTC T-3′ (forward) and 5′-CGG AAT CTA ATA TGT TAT GTT CA-3′ (reverse) [22].

PCR amplifications were carried out in 50 µl reactions using 2 µl of total genomic DNA, 1X PCR buffer, 2.5 mM MgCl2, 0.25 mM of each deoxynucleoside triphosphate, 0.4 µM of each primer, and 0.03 U/µl AmpliTaq polymerase (Applied Biosystems, Thermo Fisher Scientific). The PCR conditions for *Pfdhps* were a partial denaturation at 95 °C for 7 min, and 40 cycles with 30 s at 95 °C, 30 s at 50 °C and 1 min extension at 68 °C, and a final extension step of 5 min at 68 °C. For *Pfdhfr*, the conditions were a partial denaturation at 95 °C for 7 min, and 40 cycles with 1 min at 95 °C, 1 min at 54 °C and 2 min extension at 72 °C, and a final extension of 10 min at 72 °C. For *Pfk13* gene, the PCR conditions were: a partial denaturation at 94 °C for 4 min, and 36 cycles with 1 min at 94 °C, 1 min at 53 °C and 2 min extension at 72 °C, and a final extension of 10 min at 72 °C. Negative control (nuclease-free dH2O as a template) and positive control (*Pf* DNA) were included in each batch of PCR. PCR products from each reaction (50ul) were resolved using 1% agarose electrophoresis, excised from the gel, and purified using the QIAquick® Gel extraction kit (Qiagen, GmbH, Hilden, Germany). Purified PCR products were directly sequenced for both strands using an Applied Biosystems 3730 capillary sequencer. All *Pfk13* sequences obtained in this study were deposited in GenBank under the accession numbers MT130102 to MT130200.

### Evaluation of *Pfdhps* and *Pfdhfr* allele frequencies

After thorough inspections of each electropherogram, mutations associated with drug resistance genes (*Pfdhps* and *Pfdhfr*) or artemisinin delayed parasite clearance resistance *Pfk13* gene were identified and recorded. First, frequency of each (a) point mutations, (b) allele in *Pfdhps* and *Pfdhfr* genes, as well as (c) the combination of *Pfdhps* and *Pfdhfr* multilocus genotypes were estimated, dividing the total of point mutations/allele/combination of multilocus genotypes by the total of the samples (N) per year that successfully amplified, which corresponds to the frequency of patients with parasites that have a specific codon or allele. However, given the mixed infections found in these samples, the frequencies do not add 1. Additionally, via inspecting multiple peaks at the mutations of *Pfdhps* and *Pfdhfr* that were associated with drug resistance, polyclonal *P. falciparum* infections were identified, and their corresponding frequencies were estimated using the samples collected in the three periods (2005, 2010, and 2017/18). Statistical comparisons in the prevalence of all SNP mutations in *dhps* and *dhfr* genes in samples collected between the 2005 and 2017/18 surveys were performed using Fisher's exact tests. Statistical significance was defined by a two-sided *p* value < 0.05.

## Results

### *Pfdhps* and *Pfdhfr* allele frequencies and *P. falciparum* polyclonal infections

**Fig. 1:**
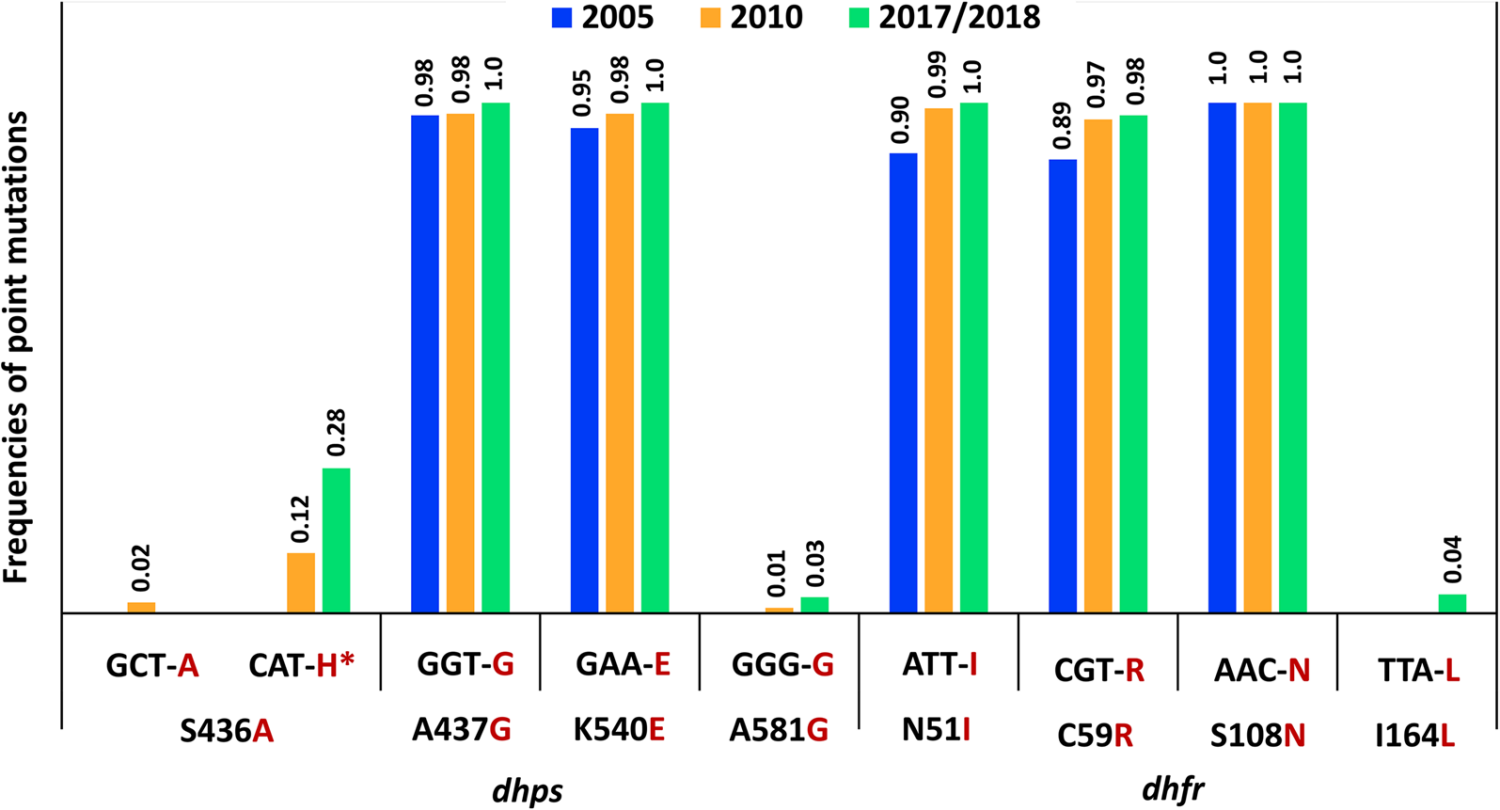
Frequency of point mutations found in *Pfdhps* and *Pfdhfr* genes. Frequencies were estimated using samples collected from pediatric malaria patients in Siaya (Western Kenya) during three periods: 2005, 2010, and 2017/18

Figure 1 shows the frequencies of genotypes at each codon for *Pfdhps* and *Pfdhfr* genes. *Pfdhps* mutations at codons 437 and 540, i.e., A437G and K540E, were detected in more than 95% of all sampled years (frequency > 0.95). However, mutation S436A was found at a low frequency (0.02, two patients) only in 2010, while the mutation S436**H** [19] seemed to significantly increase in frequency from 0.12 in 2010 to 0.28 in 2017/18 (*p* value < 0.05, Fig. 1). The A581G mutation was also present at a low frequency in 2010 (0.01, one patient) and 2017/18 (0.03, three patients; Fig. 1), and mutation A613T was not detected in any of these groups of samples. In the case of *Pfdhfr*, only the N51I, C59R, and S108N mutations were found at high frequency (> 0.85) in all sampled years. The presence of I164L mutation was only detected in three patients (frequency of 0.04, Fig. 1) in 2017/18.

**Fig. 2:**
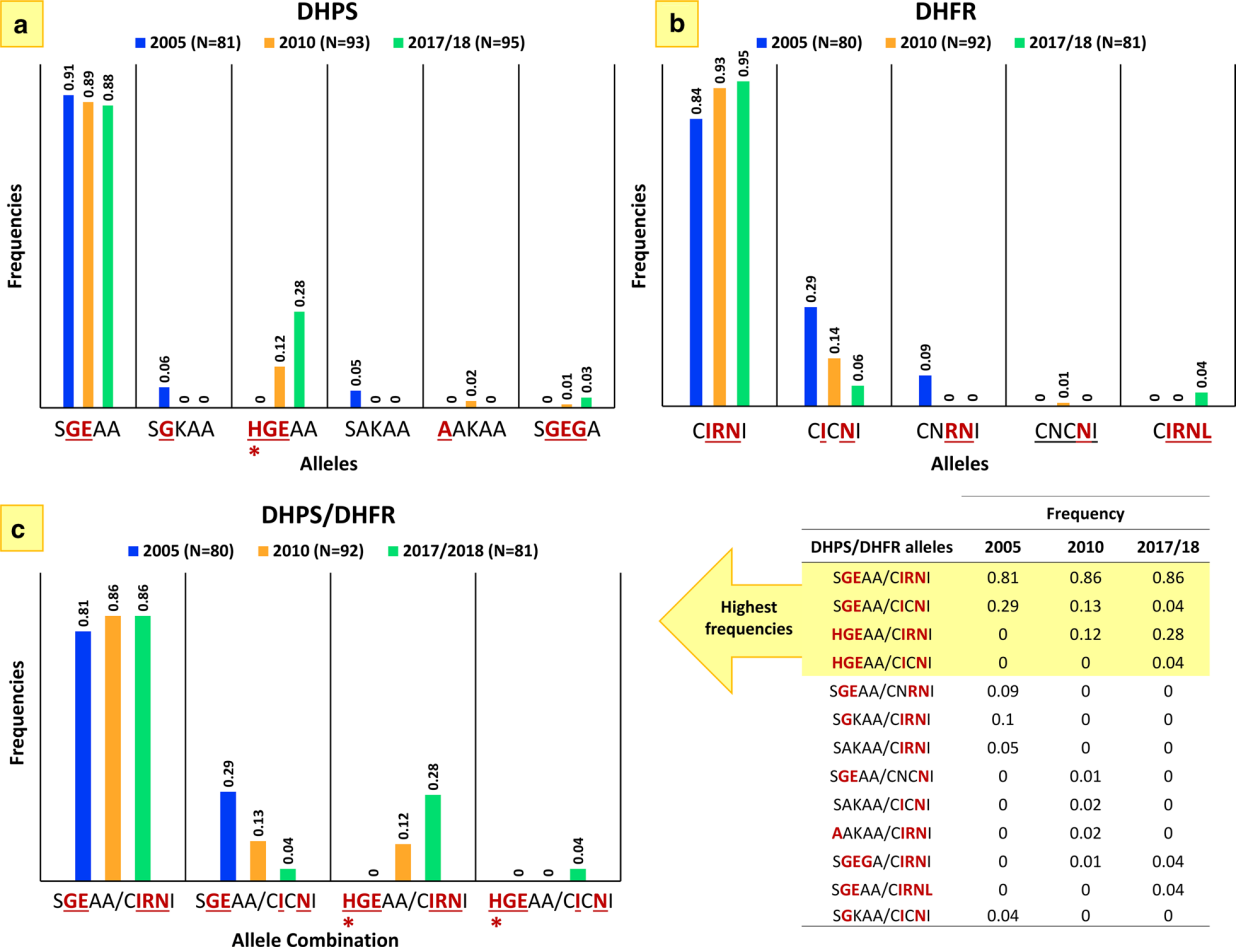
Frequency of **a** *Pfdhps* and **b** *Pfdhfr* alleles as well as **c** combined *Pfdhps/Pfdhfr* alleles. Frequencies were estimated using samples collected from pediatric malaria patients in Siaya (Western Kenya) during three periods: 2005, 2010, and 2017/18

The frequencies of *Pfdhfr* and *Pfdhps* alleles, estimated by dividing the number of each *Pfdhps* and *Pfdhfr* alleles by the total of the samples (N) per year that successfully amplified, are shown in Fig. 2. *Pfdhps* S**GE**AA (Figs. 2a) and triple mutant for *Pfdhfr* C**IRN**I (Fig. 2b) are the most frequent alleles across the sampled periods in this study population. The *Pfdhps* **HGE**AA allele, having a novel mutation S436**H**, appears to significantly increase in frequency between 2010 and 2017/18 (*p* value < 0.05, Fig. 2a). Mutations associated with SP resistance in *Pfdhps* and *Pfdhfr* genes revealed 13 *Pfdhps*/*Pfdhfr* multilocus genotypes, with frequency changing through time during the sampled periods (Fig. 2c). S**GE**AA/C**IRN**I and S**GE**AA/C**I**C**N**I were the most frequent multilocus genotypes for all sampled years; however, S**GE**AA/C**I**C**N**I seems to be significantly decreasing over time (*p* value < 0.05, Fig. 2c). Interestingly, the multilocus genotype **HGE**AA/C**IRN**I was significantly increasing in frequency between 2010 and 2017/18 (*p* value < 0.05, Fig. 2c).

Polyclonal infections detected by *Pfdhps* and *Pfdhfr* SNPs were found via examination of electropherograms. The frequency of the polyclonal *Pfdhps* infections significant increased to 0.2 in 2017/18 from 0.09 (in 2005) and 0.06 (in 2010) (*p* value < 0.05). It is worth noticing that 84% of these polyclonal infections have the novel *Pfdhps* mutation S436**H** (CAT substituted the codon TCT) in 2017/18. Since the *Pfdhps* mutation S436**H** is increasing in frequency and polyclonal infections are detectable by the polymorphism present at the sampled SNPs (*Pfdhps* and *Pfdhfr* in this case), it is not surprising that most of the *Pfdhps* S436**H** alleles are part of polyclonal infections. In contrast, the frequency of the polyclonal infections in *Pfdhfr* gene significantly decreased gradually from 0.23 in 2005 to 0.12 in 2010 and 0.05 in 2017/18 (*p* value < 0.05); this shows the fixation of the C**IRN**I allele.

### *Pfk13* population analyses

Upon inspecting sequences of the *Pfk13* propeller domain in 98 samples collected in 2017/18, none of the mutations associated with the delayed parasite clearance phenotype were found [14, 22, 23]. However, in the *Pfk13* propeller region, a nonsynonymous substitution at codon A_GCT_578S_TCT_ (2.04%, 2 patients), a nonsynonymous substitution at codon V_GTT_637I_ATT_ (2.04%, two patient), a synonymous substitution at the same codon V_GTT_637V_GTA_ (1.02%, one patient), and a nonsynonymous substitution at codon E_GAA_642D_GAT_ (2.04%, two patients) were detected in the paediatric malaria patients from Siaya (Western Kenya).

## Discussion

Molecular surveillance is considered a valuable tool to monitor the prevalence of mutations that may affect the efficacy of anti-malarial drugs [24–27]. In the context of IPTp, following the dynamic of mutations conferring resistance to SP is critical because it is the only drug approved for use in pregnant women [3, 15].

**Fig. 3:**
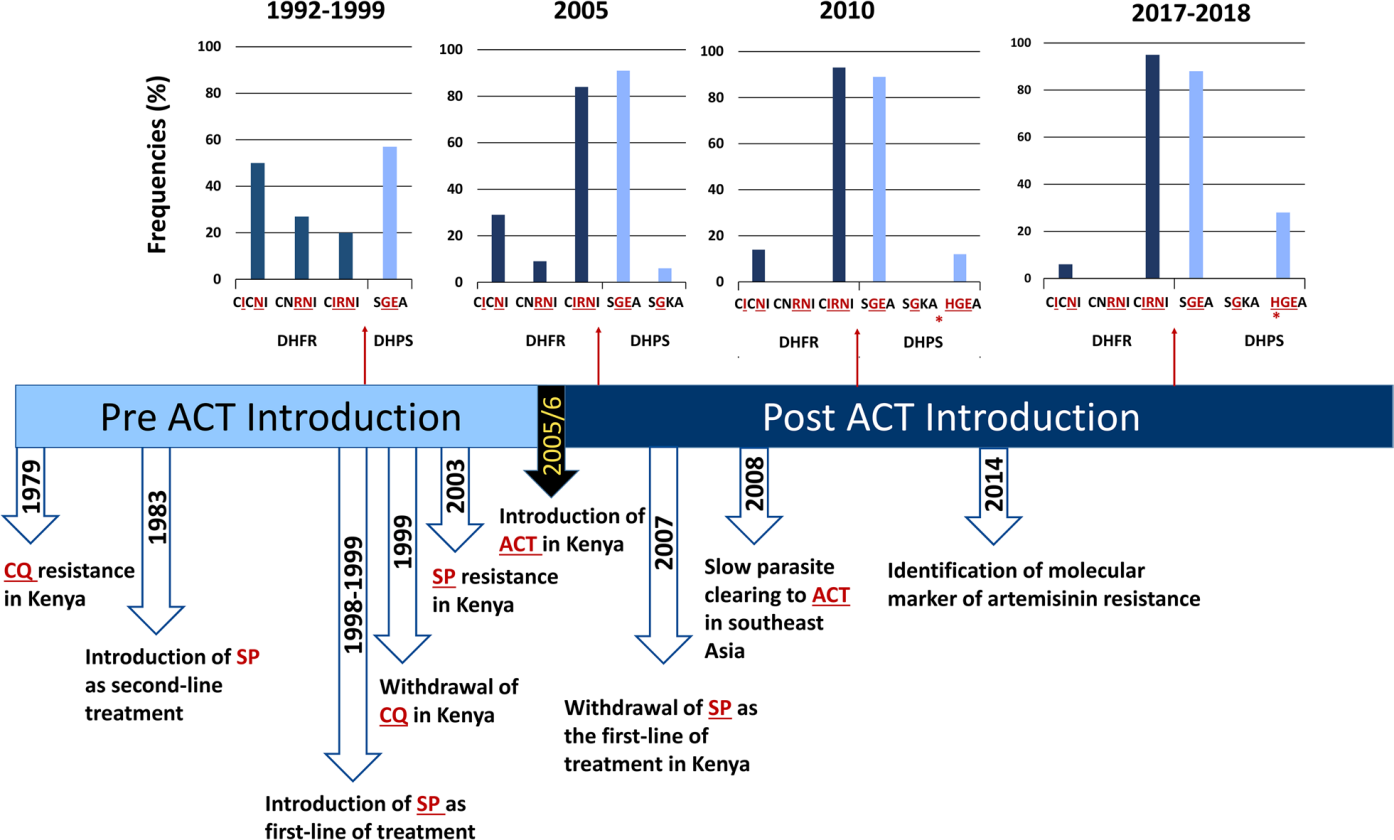
Temporal sequence describing SP use as an anti-malarial drug in Kenya and prevalence of *Pfdhps* and *Pfdhfr* alleles with mutations conferring resistance. Frequencies of *Pfdhps* and *Pfdhfr* alleles with mutations conferring resistance from 1992–1999 were previously reported by McCollum et al. [28]

This study found that the quintuple *Pfdhps*/*Pfdhfr* mutant (the *Pfdhps* double mutant A437G, K540E allele together with a *Pfdhfr* triple mutant N51I, C59R, S108N allele) associated with clinical SP treatment failure [12], remained high (0.86) in Siaya (Western Kenya) in 2010 and 2017/18 (Fig. 2c), more than a decade after the withdrawal of SP in Kenya (Fig. 3). The increase in *Pfdhfr* triple mutants is linked to a decline in the double mutant *Pfdhfr* alleles in the population, evidenced by the absence of the C59R, S108N allele in the 2010 and 2017/18 samples (Fig. 2). These patterns are consistent with the prediction made using 1992–1999 samples that allowed estimates of the relative fitness of these resistant alleles assuming drug pressure [28]. The triple *Pfdhfr* mutant that conferred higher resistance was found to have higher fitness than the two double mutant alleles [28]. Furthermore, the double *Pfdhfr* mutant alleles N51I, S108N showed a higher fitness than double mutant alleles C59R, S108N [28]. Thus, the less fit *Pfdhfr* allele under drug pressure (*i.e.*, the allele of C59R, S108N) is absent in the more recent samples.

Although the frequency of *Pfdhps* allele A437G, K540E was similar during the sampled years (0.91 in 2005 to 0.88 in 2017/18), there is an increase in the frequency of a triple mutant allele S436**H**, A437G, K540E (0.28), which has the mutation at codon 436. This mutation has been previously reported in low frequency in pregnant women from Nyanza Province (located in Western Kenya, covering the area of nowadays six counties, including Siaya county) between 2002 and 2009: 2.3% in 2002–2008 and 3.8% in 2008–2009 [19]. Although the results presented here are consistent with a scenario that positive directional selection is playing a role in the frequency increase of this new allele (S436**H**, A437G, K540E) in Western Kenya, the phenotypic effect of the *Pfdhps* S436**H** mutation to clinical drug resistance has not been determined. Thus, whether the results observed here relate to actual anti-malarial drug pressure or other processes is difficult to ascertain.

SP was the second-line anti-malarial drug until 1998 when it became the first-line malaria treatment [29]. Due to the increased frequency of chloroquine treatment failures, there was a growing SP drug pressure that may have led to the observed high prevalence of *Pfdhfr* mutations in 1998 [29]. Then, the increased use of SP selected for highly resistant mutations in *Pfdhps* [29]. By 2004, just after 5 years of SP usage in Kenya, the widespread treatment failures prompted a change in the malaria treatment drug policy to ACT in Kenya, like other African countries [30]. However, ACT did not have widespread distribution at many of the health facilities in Kenya until mid-2006. Considering the timeline described previously, the observed trends in *Pfdhfr* and *Pfdhps* mutations were unanticipated because SP has not been a first-line anti-malarial treatment in Kenya for almost 15 years.

Unlike mutations linked to chloroquine resistance, SP resistant mutations have shown to be resilient in Africa even after the drug is no longer the first-line anti-malarial treatment [13, 30, 31]. A possibility in Africa is that mutations conferring resistance to SP may not have a relative fitness cost because of the lack of wild-type alleles in the population that can outcompete the resistant ones in the absence of drug pressure, as has been suggested in South America [32, 33]. However, the significant increase in the frequencies of the *Pfdhfr* triple mutant and the *Pfdhps* allele with the S436**H** mutation is consistent with selective drug pressure. This drug preassure can be explained, at least in part, by the fact that 56% of pregnant women in Kenya took at least two SP doses in the context of IPTp in 2018, as reported by the Maternal & Child Survival Program from USAID [34].

A factor to consider is the ongoing treatment of HIV/AIDS patients with cotrimoxazole, a bacterial *dhfr*/*dhps* inhibitor used to treat respiratory tract infections and to prevent opportunistic infections. The use of cotrimoxazole in the population may have played a role in the increased frequency of mutations linked to SP resistance in malarial parasites [35] and should be considered now. There were reports showing cross-resistance of *P. falciparum *in vitro to cotrimoxazole with pyrimethamine and sulfadoxine [35, 36]. However, a recent study showed that cotrimoxazole remains effective in controlling malaria infection despite the high prevalence of SP-resistant parasites, and its use does not select for mutations associated with SP resistance [37]. Thus, at this point, the use of cotrimoxazole is not a plausible selective force that can explain the pattern observed in *Pfdhfr* and *Pfdhps* mutations.

In the case of *Pfdhps* A581G and A613T mutations associated with high-level SP resistance, they have been observed in Africa [28, 38–41], South America, and Southeast Asia [9, 10, 42, 43]. However, these mutations were detected in low frequency in the samples included in this study, and the results presented here are consistent with previous reports from Western Kenya [13, 28, 30, 40].

ACT is the first-line anti-malarial treatment in holoendemic *P. falciparum* malaria-endemic nations, including Kenya. As a result, there is ongoing molecular surveillance aimed to detect *Pfk13* mutations linked to delayed parasite clearance [1, 20]. Up to now, there is still no report on the presence of a delayed parasite clearance phenotype for ACT in Kenya. Mutation A578S, found in this study, is the most predominant mutation in sub-Saharan Africa [23, 44–48] and has been reported in both pre- and post-ACT parasites, with frequencies between 1.2 and 10% in samples from different malaria ecological zones in Kenya [48, 49]. For example, A578S mutation has a frequency of 2.8% in samples from Kisumu (Western Kenya) [45]. These results support the notion that A578S is the most widespread *Pfk13* SNP observed in Africa, including countries such as Mali, Angola, Democratic Republic of Congo, Uganda, Gabon, Ghana, and Kenya [20, 22, 23, 45, 47, 48, 50–52]. However, in all the studies, this mutation was detected at low frequencies. This finding is consistent with the proposed model that many of these *Pfk13* mutations are slightly deleterious and maintained as the *P. falciparum* population expanded, making selection less efficient [22]. Although the functional impact of A578S in terms of ACT efficacy remains unclear, recent studies have hinted at a potential effect. In particular, A578S is very close to the C580Y mutation, and molecular modelling and mutational sensitivity prediction performed by Mohon et al*.* [52] suggested that the A578S SNP could disrupt the function of the propeller domain. Nevertheless, experimental evidence is still lacking [20, 53].

## Conclusion

Although the evolutionary processes driving the observed pattern remain elusive because of a lack of specific phenotypic information on the S436**H** mutation, its increase in frequency seems consistent with drug pressure. SP is no longer the first-line anti-malarial drug in Kenya, but it is still widely used as part of IPTp [15]. More studies are warranted to discern whether the *Pfdhps/Pfdhfr* multilocus mutant (S436H, A437G, K540E/ N51I, C59R, S108N) adversely impact the efficacy of SP in IPTp in the context of drug efficacy evaluations. Such information is critical, considering that SP in IPTp remains an essential tool for reducing disease burden in sub-Saharan Africa [15]. On a separate note, there is no evidence indicating that *Pfk13* mutations linked with the delayed phenotype were present in Kenya when these samples were collected. However, sustaining the molecular surveillance is important since there are reports in China of an imported malaria case from Rwanda with the mutation R561H linked to artemisinin resistance [54].

## Data Availability

Sequences were deposited in the GenBank with the accession numbers MT130102 to MT130200.

## Abbreviations

ACT: Artemisinin-based combination therapy
CBC: Complete blood counts
DHFR: Dihydrofolate reductase
DHPS: Dihydropteroate synthase
IPTi: Intermittent preventive treatment in infants
IPTp: Intermittent preventive treatment in pregnancy
ITNs: Insecticide-treated bed-nets
SP: Sulfadoxine-pyrimethamine
WHO: World Health Organization
